# Why we do need explainable AI for healthcare

**DOI:** 10.1186/s41512-025-00209-4

**Published:** 2025-12-02

**Authors:** Giovanni Cinà, Tabea E. Röber, Rob Goedhart, Ş. İlker Birbil

**Affiliations:** 1 Department of Medical Informatics, Amsterdam University Medical Center, Amsterdam, The Netherlands; 2 Institute of Logic, Language, and Computation, University of Amsterdam, Amsterdam, The Netherlands; 3 Department of Business Analytics, Amsterdam Business School, University of Amsterdam, Amsterdam, The Netherlands

**Keywords:** Medical AI, Explainable AI, Clinical applications

## Abstract

The recent uptake in certified Artificial Intelligence (AI) tools for healthcare applications has renewed the debate around their adoption. Explainable AI, the sub-discipline promising to render AI devices more transparent and trustworthy, has also come under scrutiny as part of this discussion. Some experts in the medical AI space debate the reliability of Explainable AI techniques, expressing concerns on their use and inclusion in guidelines and standards. Revisiting such criticisms, this article offers a balanced perspective on the utility of Explainable AI, focusing on the specificity of clinical applications of AI and placing them in the context of healthcare interventions. Against its detractors and despite valid concerns, we argue that the Explainable AI research program is still central to human-machine interaction and ultimately a useful tool against loss of control, a danger that cannot be prevented by rigorous clinical validation alone.

## Introduction

Along with the surge of artificial intelligence (AI) and the accompanying increase in model size and complexity, there has been a spike of interest in Explainable AI (XAI henceforth), namely AI that allows humans to understand its inner workings [1–5]. Especially in safety-critical domains such as healthcare, it is perceived that XAI can engender trust, help monitoring bias, and facilitate AI development [1, 6]. For an example of an interpretable model intended for clinical use, Letham et al. [7] developed a stroke prediction model matching the performance of the most accurate machine learning algorithms, while remaining as interpretable as conventional scoring methods used in clinical practice.

Despite the enthusiasm, the insertion of AI transparency requirements into the law of some countries (see e.g., the European AI Act [8]), and a growing community of researchers devoting energy to XAI, there is currently no consensus on the reliability of XAI techniques, and several researchers have cast serious doubts on whether XAI solutions should be incorporated into guidelines and standards, or even deployed at all [9, 10].

Undoubtedly, there are conflicting desires: on one hand we want machines performing better than humans, and on the other hand we desire machines providing human-understandable explanations. The sub-symbolic character of statistical learning techniques powering AIs' super-human capacities-–such as the ability to juggle dozens or hundreds of factors-–also contributes to rendering machine learning models opaque to humans. Trying to overcome this challenge, XAI techniques may do more harm than good, confusing or even misleading human users.

What role is left then for XAI in the medical realm, where adoption and trust of new technologies are notoriously complex? Given the wealth and heterogeneity of XAI techniques, as well as the doubts and limitations raised by researchers, the answer to this question is not straightforward, and the literature lacks a systematic discourse of the key arguments.

In our work, we address that gap by collecting, reviewing, and appraising seven important criticisms that are leveled against XAI. Our contribution is a debate of said criticisms—offer both arguments and counterarguments for each—in the context of clinical application of AI technologies. We conclude that, despite several critical points of attention, there is no decisive argument to abandon the search for better XAI techniques, especially in the medical field, where a higher level of oversight is paramount.

## Background

There is a wide variety of techniques for XAI, which has become an umbrella term encompassing interrelated but distinct activities. Techniques can roughly be categorized into local vs. global, and model-specific vs. model-agnostic approaches. Local methods aim to explain model outputs for individual samples, while global methods focus on making models more explainable at an aggregate level. Model-specific methods are tailored to explain a specific type of model, while model-agnostic methods can be applied to a range of different models. Many of the well-known techniques yield post-hoc explanations, meaning that they generate explanations for already trained, so-called ‘‘black-box’’, models. Furthermore, some recent research advocates for ‘‘mechanistic interpretability’’—which aims to dissect the model by determining which sub-parts of a model are responsible for certain skills or behaviors (in other words, functionally relevant components)—as the preferable approach. Alternatively, there exist approaches that are inherently explainable, also known as white-box models, such as decision trees and linear regression models. A detailed taxonomy is beyond the scope of this paper; for an extensive overview, we refer the reader to existing reviews [11–14].

What is important to stress is that, in order to overcome the opaqueness of black-box models, XAI techniques often bring in additional layers of complexity. For example, explanations themselves can be inaccurate or deceiving [10, 15], and different explanatory techniques may not yield consistent results [16]. Besides, since an objective ground truth is lacking, it is hard to evaluate XAI techniques [17, 18], and oftentimes, there is a trade-off between explainability and other desiderata like high performance [19, 20]. Lastly, privacy concerns are heightened in the light of explanation methods as explanations could give away information that may be used to infer whether a sample belongs to the training data [21].

## Criticisms to Explainable AI

**Fig. 1:**
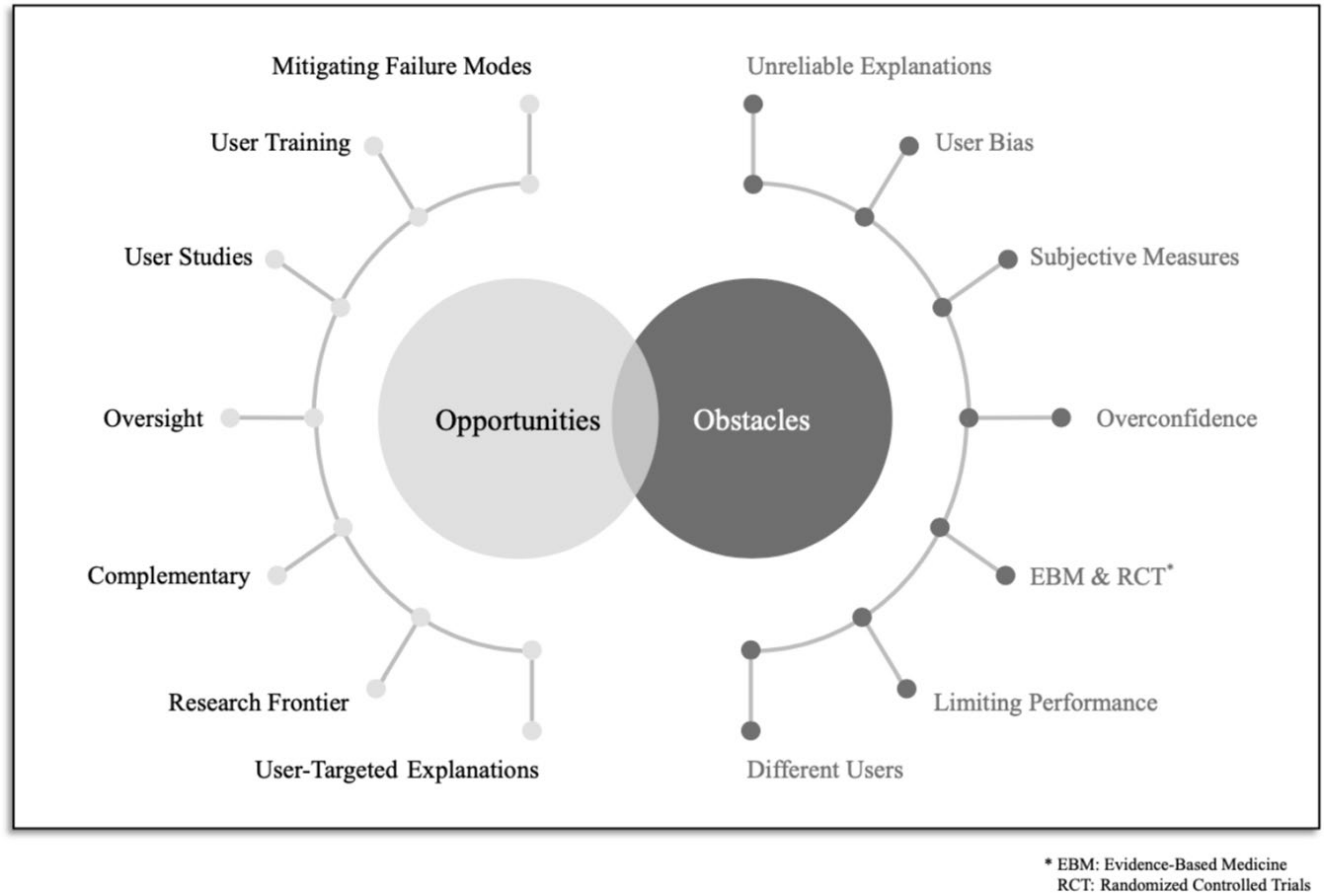
A visual summary of the criticisms and the related opportunities

We collect and discuss the main doubts raised about the usefulness and efficacy of XAI, in no particular order. The criticisms, along with the main counterpoints discussed below, are visually represented in Fig. 1. As the figure suggests, even though the criticisms underscore the existence of obstacles in the use of XAI, the counterpoints suggest how these challenges can be overcome or mitigated by directing research focus.

- XAI techniques may be incorrect or unreliable.
- Human users may fall prey to biases when interacting with XAI tools.
- There are no objective measures of explainability.
- XAI may work on an aggregate level, but not on an individual level.
- Evidence-based assessment is the proper way to evaluate medical (AI) interventions, not XAI.
- In the medical domain, we cannot trade performance for explainability.
- XAI is not suitable for all users.

It should be noted that some of these points share a thematic affinity and some overlap, as will become apparent during the discussion. Points 1 and 3 for example, are of a technical nature, pertaining to approximation and metrics, while points 2 and 7 are related to the user side of the equation, namely to cognitive traps and modes of utilization that human users may have. Our formulation is therefore not a strict categorization but rather a handle on how to debate various criticisms on XAI.

### XAI techniques may be incorrect or unreliable

**Criticism.** XAI techniques are sometimes faulty: they can be fooled with adversarial attacks or with noise [22–25]. Moreover, for XAI techniques that approximate an original black-box model, there can be an additional discrepancy between the explanation and the real model behavior, resulting in unreliable explanations (sometimes referred to as lack of faithfulness) [15].

**Discussion.** While these points are true, the mitigating factor here is that every technology has failure modes. As long as there is awareness and know how to counter undesired effects, such techniques can still be useful. Furthermore, some such negative results can also be a pointer on how to improve XAI techniques. For instance, SHAP (one of the techniques offering individual-level explanations [26]) is vulnerable to adversarial attacks [22]; in this case, the recommendation is that out-of-distribution samples should not be employed in approximate SHAP calculations. This said, whenever possible one should prioritize using a simple white-box model. Finally, it is also worth noting that there is ongoing work in the estimation of uncertainty of explanations (e.g., uncertainty of Shapley values [27]) which may also aid in assessing their reliability.

### Human users may fall prey to biases when interacting with XAI tools

**Criticism.** Users of XAI techniques struggle to understand what a good explanation is and often fall prey to biases—e.g., believing more readily explanations that confirm their own beliefs, the so-called confirmation bias—or misuse XAI tools [28–30]. Efforts have been made in recent years to identify different types of cognitive biases and their interaction with XAI systems, see e.g. [31].

**Discussion.** Such remarks are on point and should be addressed when designing interfaces of AI tools and training programs, but they do not constitute a sufficient reason to give up on XAI. It is very well documented that many researchers misunderstand and misuse *p*-values [32], but this is not a good argument to abandon significance testing. A similar point could be made about conventional medical procedures, where different kinds of bias have been documented [33]. The best solution for such problems is to have the users properly trained; notably, when it comes to medical devices, the training of users is quite well regulated; e.g., in Europe via the Medical Device Regulation and the ISO standards [34]. Recent efforts show an increase in research on how cognitive biases are affected by and affect XAI interactions; review papers like Bertrand et al. [31] map out the landscape of different biases, helping to address them in future research as well as in the development and testing phases of AI-based decision support tools. Alternatively, the field should develop techniques to de-bias explanations whenever possible [35].

### There are no objective measures of explainability

**Criticism.** There is no obvious ground truth for explainability, and therefore there is no straightforward way to assess how good an XAI technique really is [17, 18].

**Discussion.** This surely is a barrier, and more work is required to establish how the reliability of XAI tools can be tested; what is a good explanation, and what is useful to the user/developer/regulator are questions that have an intrinsic psychological nature and would benefit from more principled and widespread experimentation. Several of these concerns have been raised recently [36], and substantial work has already been done in investigating what constitutes an appropriate and useful explanation [22, 37–40]. These efforts include eliciting users’ preferences with user studies, and deriving insights on the notion of explainability from the perspective of social sciences and related fields. Observing this need for an interdisciplinary structure, many scientists have recently participated in projects, workshops, and discussion groups that involve colleagues working in diverse areas. Such interdisciplinary efforts may help in creating benchmarks that could be used to test XAI techniques more systematically.

### XAI may work on an aggregate level, but not on an individual level

**Criticism.** Ghassemi et al. [10] distinguish XAI techniques used in the specific (individual level) case and in the general case, finding that they can be of use in the latter but not in the former. The reason is that users too often cannot decide if the explanation is sensible for an individual sample and have to base their judgment on an unwarranted intuition.

**Discussion.** While this risk is concrete, it is important to realize that this criticism mostly applies to certain types of data. In images, single input features (i.e., pixels) have no intrinsic meaning; only certain patterns of feature activation have meaning. When a certain area of an image is highlighted we simply do not know if what we recognize (e.g., the shape of a kidney) is the same as what the AI recognizes, because we have no access to the high-level representations of the machine. Hence, we cannot be sure whether the machine is recognizing the kidney as the salient part of the image at hand, or if there is some other spurious reason. There are however data types, like Electronic Health Records, where single input features have a very clear semantics. If, for instance, the patient’s values of BMI and glucose are singled out as salient by an explainability technique, this may be enough for a clinical user to consider whether the AI’s output is sensible [41].

Thus, in some applications, having XAI developed on an individual level in a way that allows users to ‘‘check’’ the reason(s) behind the prediction, can be a way to find and escalate possible problems of the AI at an aggregate level. For instance, recent work by Cinà et al. [35] aims to address this issue. Especially when the data and the AI environment are of high complexity, we need oversight mechanisms to continuously monitor and detect unforeseen failure modes, and individual users can play a role in such monitoring processes. Furthermore, the explanations of AI predictions will typically be one of the pieces of information that clinicians take into account. In current practice, for example, physicians obtain results of different tests, which at times may even be potentially conflicting, e.g., radiopaque lesion resembling infectious mass in a CT scan but lack of elevated infection markers. Such different factors are weighed in, each with its degree of uncertainty, to arrive at a decision. In this workflow, the input(s) of XAI techniques can be contrasted with other sources of information and be instrumental in achieving a deeper insight into the clinical case at hand and decide whether to trust the AI. Notably, this may also mean that the clinicians do not trust the AI’s prediction on a specific patient because the AI has shown that, e.g., some lab results are no problem while in reality the clinicians finds them worrying.

### Evidence-based assessment is the proper way to evaluate medical interventions, not XAI

**Criticism.** According to this criticism, randomized controlled trials (RCTs) and evidence-based assessment are better ways to evaluate medical interventions than XAI. The primary goal is to have interventions that work, even if we do not understand why. Black-box solutions are used in medicine already, so we do not need XAI as long as we have established that a tool or intervention is effective. The desire for explainability is secondary [9].

**Discussion.** It is indisputable that XAI should not be conceived as a replacement of evidence-based instruments to validate model effectiveness. XAI techniques are in general not sources of evidence on causal effects (e.g., treatment effects), but only a way to unpack model behaviour, which will have a causal interpretation only if the model is causal. However, the criticism raises a false dichotomy. We do not need to choose between a thorough evaluation and an explainable model: thorough evaluation should be a default (and it often is, given current regulations), and the need for explainability can be discussed separately.

Furthermore, framing the discourse about explainability as a binary classification between primary and secondary requirements is needlessly reductionist. Surely, there can be trade-offs with accuracy that we must be cognizant of (more on this later), but the benefit of explainability may pay off in other areas, e.g., the discovery of biased outcomes for certain subgroups, or the disentangling of causal effects from spurious ones. As put forward by various governing bodies, explainability is one aspect within a set of requirements that are partially interdependent and should be balanced against each other. The pursuit of explainability will have different priority depending on the application. An AI tool to speed up reconstruction of images in the back-end might not need explainability, while a tool to predict risk of complications might need it if the reasons behind the predicted risks are to be evaluated by users. A blanket statement declaring explainability ‘‘secondary’’ does not help in determining which case is which.

A second point should be made about validation of AI tools and evidence-based medicine (EBM). EBM can rely on a set of protocols that are established and widely used for the certification of new interventions. It should be noted however that, despite its indisputable importance, EBM is not a laser-precise discipline. For example, there is a vast body of literature highlighting the fact that clinical trials are often not reflecting clinical practice, or have non-representative populations, or are incomparable with each other, or become quickly outdated [42]. Furthermore, as argued by several researchers [42–45], RCTs do not always control for the biases they are intended to control, they do not produce reliably generalizable knowledge, or they can create unnecessary constraints on clinical testing. In addition, obtaining statistically significant evidence is often extremely time-consuming and expensive. Finally, the success rate of clinical trials for medications is notoriously low [46]. Against this backdrop, we should be very careful to promote the initiation of many studies or trials testing the effectiveness of tools whose inner workings are entirely unknown, i.e., black boxes.

For this very reason, explainability can be seen as a precious tool not just for the end user, but also for the developer or trialist: models may be discarded before reaching the test or RCT phase if they are found to be clinically implausible thanks to explainability techniques, or said techniques could aid in identifying which variables should be checked for balance between the RCT’s arms. In this sense, while we do not dispute the high position of RCTs in the hierarchy of evidence [47], explainability could be a complementary tool that can even help expedite or improve the effectiveness of RCTs and EBM as a whole (of course, keeping in mind the challenges discussed here).

### In the medical domain, we cannot trade performance for explainability

**Criticism.** The trade-off between explainability and performance may induce people to use simpler and less accurate models, rather than more effective ones. This is a problem, as we may be exchanging a further improvement in medical outcomes with a debatable increase in transparency. There are several medical interventions (e.g., some drugs) for which a mechanistic explanation is not known, but they are nevertheless proven to be effective and widely used. If we are accepting of black-box interventions in healthcare, we can allow effective AI models to be opaque as well [9].

**Discussion.** It is indeed the case that some explainability techniques (or simpler, more interpretable models) can result in lower performance. Whether this is desirable is a decision that should be taken case-by-case. Moreover, the analogy with medications, and the fact that we accept them being black-boxes, seems misguided. There are already different tools in medical practice for which different levels of understanding are required. For example, as opposed to drugs, radiography machines can only be operated by specialists. Hence, for complex tools we do require users to have some knowledge of the inner workings of the device. The reason underlying this choice is that there are failure modes we want to be able to control. Moreover, the fact that now we do not require explanations for some interventions (as long as they are proven to work) does not mean that this is the desirable way forward. On the contrary, in some cases this could be construed as a problem. Arguably, we should strive to understand why certain treatments are effective. Along this line of reasoning, if we can construct a tool with built-in transparency—at a bearable cost in terms of trade-offs—we should definitely pursue it.

**Fig. 2:**
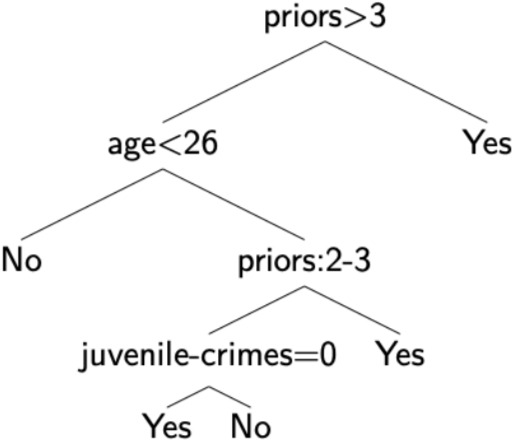
Example of an accurate white-box algorithm for the COMPAS recidivism problem (Fig. 4 in Hu et al. [48])

Finally, a ‘‘simple’’ algorithm is not necessarily less accurate. When coupled with feature engineering or feature reduction, even those ‘‘simple’’ algorithms may achieve good performances. This criticism thus overlooks an important line of work on inherently interpretable methods that aim at matching the performances of more complex models [7, 15]. Figure 2 showcases a simple model which is easy to understand and achieves high accuracy for its task.

### XAI is not suitable for all users

**Criticism.** XAI techniques can be used to support the work of different professionals interacting with an AI tool. These roles may include the developers, the medical experts, the regulators, and so on, each with different requirements and explainability needs. Arguably, XAI techniques could be useful for trained professionals such as developers or auditors, but could be dangerous when shown to untrained users.

**Discussion.** This point has some overlap with points 2 and 4, but it is nonetheless important to stress that XAI techniques will almost never offer a one-size-fits-all solution. Different kinds of interaction with the machine will be required depending on the purpose of the participating human; it is highly likely that different techniques will answer different needs [49]. Current research extends to interactive machine learning to provide user-targeted explanations [50, 51]. As to the potential misuse or misunderstanding of XAI by simple users, the solution lies in a combination of robust methodology, proper training, and expectation management.

## Should we downsize our efforts in XAI for healthcare?

We reviewed some of the core criticisms offered in the literature against the widespread use of XAI techniques in medical contexts. Some of the qualms, as well as some of the counterpoints, are not limited to healthcare applications and can be recast at a more general level. However, we should be mindful of how the debate is influenced by the higher stakes present in safety-critical AI applications in the health realm. It is worth remarking that demoting the usefulness of explainable medical AI means stifling the efforts to get human oversight over a new technology that can be deployed at scale very rapidly, potentially affecting a large swath of the population. XAI is not a silver bullet that will solve all of AI’s problems, and in particular it is not a substitute for rigorous evaluation of model performance. Multiple important efforts have been made to systematically list limitations and challenges that XAI on a more general level faces, aiming to facilitate and encourage research to adequately address those [15, 52]. Nonetheless, XAI tools can be of value in several circumstances. Human oversight over machines is still an important tenet, and the introduction of more black-boxes is not going to stem the loss of human control in a quickly digitizing world. It has been argued that augmentation of human capacities is preferable to automation [53], since the latter leads to deskilling as well as detachment and unemployment spikes. Augmentation, however, presupposes the possibility of a human-machine synergy; in other words, a way for humans to relate and engage with AI.

Different public bodies, including the European Union, are developing regulations for AI products which mention transparency requirements (see, for example, Nannini et al. [54] for an overview of regulatory policy developments regarding XAI). While the field of XAI may not yet be at a level of maturity where XAI techniques are embedded into guidelines in the form of hard requirements, it could be meaningful to allow the use of XAI techniques—without enforcing a specific choice—and require an argument of why a certain technique was chosen. Frameworks such as those proposed by Nannini et al. [24] may further guide the responsible adoption of XAI solutions in practice by assessing of potential risks related to XAI solutions before their implementation.

We noted in the introduction the inherent tension in the request for explainability: while we want machines to perform at super-human level, we also want them to be understandable by humans. While the former goal requires an increase in model complexity, the latter requires somewhat simple explanations, leaving us with the conundrum: a ‘‘simple’’ explanation might be handy for humans but hide away the complexity and potentially be misleading, while a complex explanation will do the opposite. No need to despair though; this is just another case of competing objectives, not a good reason to toss one of the objectives out the window (for another example of competing objectives: reducing healthcare costs and keeping people alive). The challenge is to find a trade-off that is satisfactory, a challenge that is extremely case-dependent. The question should be: while explainability may not be a requirement for healthcare interventions *tout court*, is AI a technology on which we think extra explainability is needed? This technology has peculiar aspects that set it apart, including the potential to be prejudiced against subgroups based on gender, ethnic background, and socioeconomic status, [55] the fact that it may lose reliability when the input data changes [56], or that it may rely on spurious correlations found in the data [57], i.e., associations that are not causal. For these reasons, coupled with the potential of rapid and impactful adoption, we argue that a higher level of oversight is preferable compared to other healthcare interventions.

## Conclusion

The research on XAI is growing very rapidly. Thanks to this growth, new techniques are tested extensively by many researchers in the field, and the duration to become a time-tested approach is much shorter than it has been in the past. Given the arguments we discussed, and the aforementioned possibility to rapidly hone useful techniques, we conclude that we should step up our efforts in XAI for healthcare, as this research program addresses a core issue.

Ultimately, while we want to make clinicians more productive with the aid of machines, we still want clinicians to be the mediators that can bridge the coldness of algorithms and scientific protocols with the human experience of the patient. These two objectives can be made compatible only if we find a way for clinicians to grasp why the machine is acting in a certain way. If clinical users of medical AI cannot interact with machines, if their role becomes that of mere executors of interventions that are proven to be effective, the sensitive task of providing care could well become de-humanizing.

## Data Availability

No datasets were generated or analysed during the current study.
